# Items

**Published:** 1905-11

**Authors:** 


### ITEMS.

The Bloodless Phlebotomist is the title of a trade periodical issued by the Denver Chemical Company in the interests of antiphlogistin. The publishers announce that they hope to make the publication a permanency.

The Palisade Manufacturing Company, of Yonkers, recently published a pamphlet entitled, "An Improved and Accurate Method of Staining Blood Films." It is illustrated by photomicrographs of stained specimens from published cases, which are not only artistic but accurate and instructive. The brochure will be sent to physicians upon application to the publishers.

The Moet et Chandon "White Seal" is an ideal champagne, not only for the table but for the sick room. Physicians will do well to bear this in mind whenever champagne is indicated in the treatment of disease or the palliation of symptoms. George A. Kessler & Company are the sole importers of this superb wine. 20 Beaver street, New York.

Messrs. H. K. Mulford Company, Philadelphia, have issued an illustrated brochure entitled, A Contribution to the Therapy of Antistreptococcic Serum. Attention is particularly directed to the fact, by this house, that the exact immunisation power of every cc. of antistreptococcic serum is accurately determined before leaving the laboratories. The color process illustrations of diphtheria in this pamphlet are worthy of praise.

The Arlington Chemical Company, Yonkers, is issuing a series of portfolios containing portraits in photogravure of historic medical personages. The first of the series is Ambrose Paré,—an artistic picture.

